# A High-Throughput RNA Extraction for Sprouted Single-Seed Barley (*Hordeum vulgare* L.) Rich in Polysaccharides

**DOI:** 10.3390/plants6010001

**Published:** 2016-12-22

**Authors:** Abdur Rashid, Thomas Baldwin, Michael Gines, Phil Bregitzer, Kathy Esvelt Klos

**Affiliations:** 1 United States Department of Agriculture—Agriculture Research Service (USDA-ARS), Small Grains and Potato Germplasm Research Unit, 1691 S. 2700 W., Aberdeen, ID 83210, USA; tom.baldwin@ars.usda.gov (T.B.); Phil.Bregitzer@ars.usda.gov (P.B.); 2 Department of Plant and Wild Life Sciences, Brigham Young University (BYU), Provo, UT 84602, USA; michael.c.gines@byu.net

**Keywords:** barley, cereals, sprouted seed, germination, high-quality RNA, single-seed extraction, qRT-PCR

## Abstract

Germinated seed from cereal crops including barley (*Hordeum vulgare* L.) is an important tissue to extract RNA and analyze expression levels of genes that control aspects of germination. These tissues are rich in polysaccharides and most methods for RNA extraction are not suitable to handle the excess polysaccharides. Here, we compare the current methods for RNA extraction applicable to germinated barley tissue. We found that although some of these standard methods produced high-quality RNA, the process of extraction was drastically slow, mostly because the frozen seed tissue powder from liquid N_2_ grinding became recalcitrant to buffer mixing. Our suggested modifications to the protocols removed the need for liquid N_2_ grinding and significantly increased the output efficiency of RNA extraction. Our modified protocol has applications in other cereal tissues rich in polysaccharides, including oat.

## 1. Introduction

Seed germination is a complex process wherein the seed absorbs moisture, breaks dormancy, breaks down the endosperm, and allows for the emergence of the radicle from the embryo. In barley, an understanding of germination is highly emphasized because of its role in the economically important malting process. Germination and malt production involve dynamic changes to enzymatic activity, such as to the glucanases, amylases, and proteinases as physiological and chemical changes are made to the starchy endosperm [1,2,3,4,5,6]. Therefore, transcript expression analysis of these related genes can provide information about the level of activity in the germination process [7,8,9].

Here, we investigated several extraction protocols developed for ample high-quality RNA for analysis of gene expression from seed, including cereal crop seed [10,11,12,13], designed to tolerate high amounts of polysaccharides to work with 3–4 day germinated seed, an important time point in further research. Although the different methods produced high-quality RNA from this type of tissue, most procedures were too slow for the high-throughput processing that is required in large scale studies. Our comparison of methods and protocol modifications provide insight into the most appropriate method for high-throughput application that does not require liquid N_2_, produces RNA of high quality, and is much faster than the source methods.

## 2. Results and Discussion

We initially tested three standard protocols for high quality RNA extraction from germinated barley seeds. Protocols included (i) the GeneJET plant RNA minikit (Thermo Fisher Scientific, Vilnius, Lithuania); (ii) the TRIzol reagent-based protocol (Zymo Research, Irvine, CA, USA); and (iii) the Li and Trick [11] protocol. Additionally, Wang et al. [12] was evaluated as it has a similar protocol as the Li and Trick [11]. Protocols were compared for RNA quality as measured by spectrophotometry at A260/280 and A260/230 ratios (Table 1) before and after a DNase I treatment. An acceptable value of RNA quality was considered as greater than 1.8 [14].

**Figure 1:**
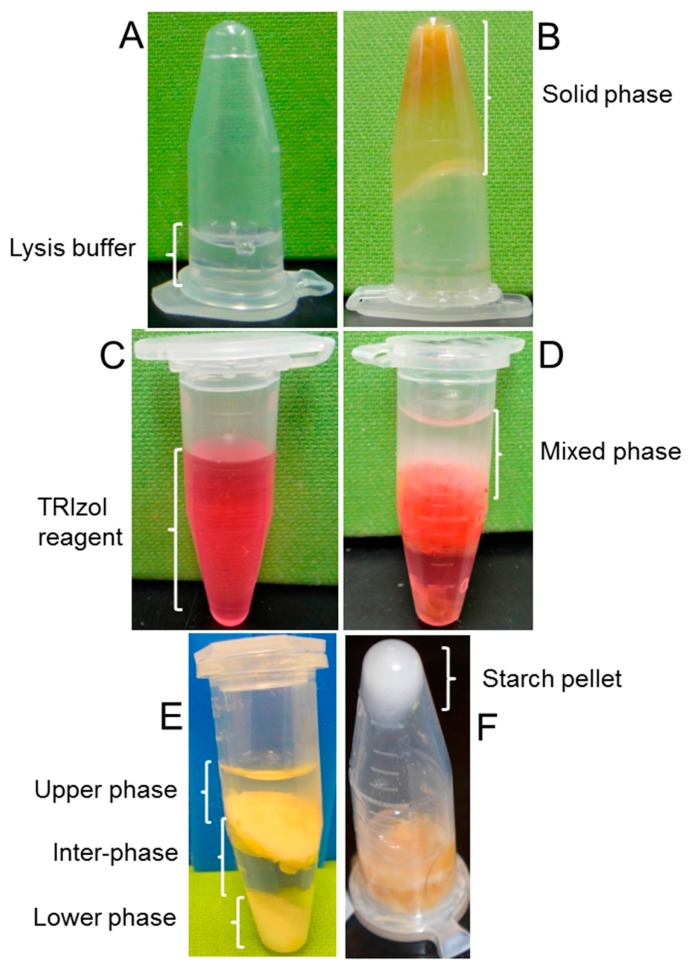
RNA extraction from 4-day sprouted seed. *Upper panel*: homogenate solidification during RNA extraction by GeneJet minikit using the standard protocol. (**A**) An inverted microcentrifuge tube showing lysis buffer at the bottom; (**B**) a tube showing the solidified homogenate. *Middle panel*: incomplete phase separation in the standard TRIzol method; (**C**) a microcentrifuge tube containing TRIzol reagent; (**D**) a tube showing the hazy phase separation of the homogenate. *Lower panel:* expected phase separation in the Li and Trick [11] method; (**E**) a microcentrifuge tube showing an upper aqueous phase, an interphase (phenol-chloroform phase) in the middle, and a lower solid phase at the bottom; (**F**) a tube in inverted orientation following removal of the upper aqueous phase showing shiny starch granules deposited at the bottom.

The GeneJet minikit failed to extract RNA due to homogenate solidification that prevented phase separation. Despite following the manufacturer’s protocol for handling polyphenols, terpenoids or polysaccharides in samples, homogenate remained solidified (Figure 1B). The standard TRIzol method produced incomplete and hazy phase separation of the homogenates, even after prolonged centrifugation (Figure 1D), and the resulting RNA from the upper aqueous layer was poor in terms of quality and yield (Table 1). In contrast, we obtained the expected phase separation of the homogenates using the standard Li and Trick [11] method such that an upper aqueous phase containing nucleic acids, an interphase/phenol-chloroform phase in the middle, and a solid shiny phase at the bottom containing the sediment of the starch granules were clearly delineated (Figure 1E,F).

**Figure 2:**
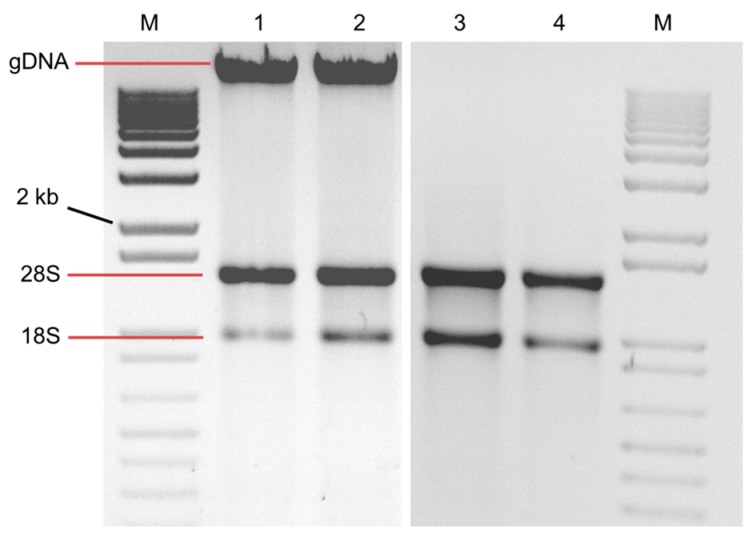
Agarose gel electrophoresis of RNA extracted from sprouted seed by the modified Li and Trick [11] method. M: 1kb marker; lanes 1–2: before DNase treatment; lanes 3–4: after DNase treatment; gDNA refers to genomic DNA.

**Figure 3:**
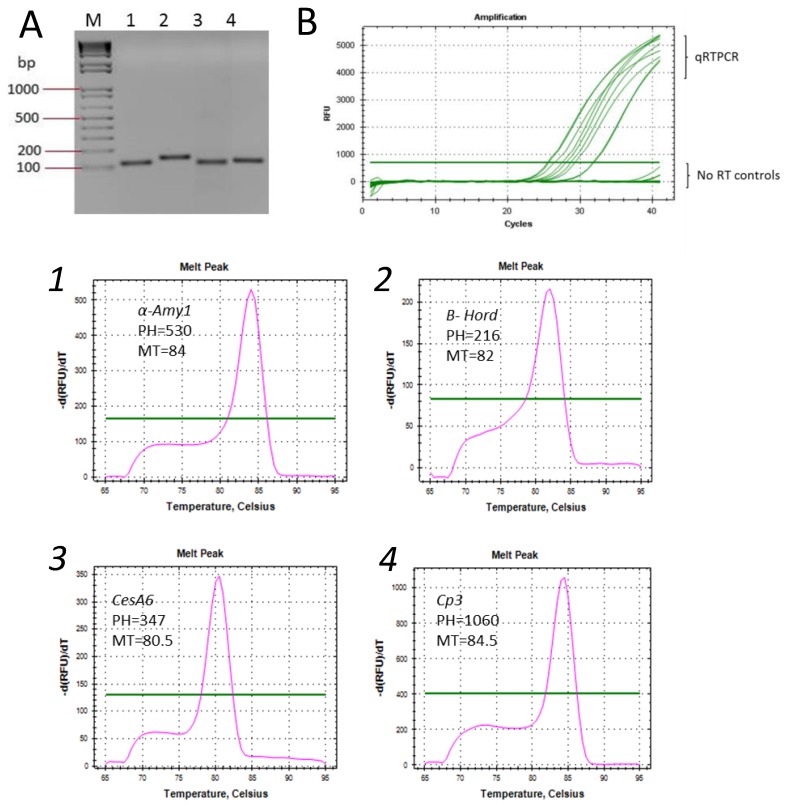
Reverse transcription PCR (RT-PCR) and quantitative real-time PCR (qRT-PCR) assays. (**A**) an agarose gel; and (**B**) Ct values showing PCR amplification of four gene fragments from cDNA synthesized from RNA extracted by the modified Li and Trick [11] method and no reverse transcriptase (RT) controls. Lanes, (M): 100 bp marker; (**1**) α-*amy1*; (**2**) *B-hord*; (**3**) Ces*A6*; (**4**) *Cp3*; and qRT-PCR melt curves of the above genes (corresponding numbers). PH: peak height; MT: melting temperature; green horizontal lines indicate threshold levels.

The use of liquid N_2_ to grind samples is one commonality among these published methods. However, we found that the frozen powder resulting from liquid N_2_ grinding became recalcitrant to the buffer even after prolonged and vigorous vortexing. In addition, the grinding step is slow and adds a freeze/thaw component to the procedure that could degrade RNA. We circumvented this time-consuming step by grinding the soft sprouted seed tissue at room temperature directly in the extraction buffer. Although the quality and yield of RNA resulting from the standard vs. modified method of Li and Trick [11] (or of Wang et al. [12]) were not significantly different after purification (Table 1), the protocol modification enabled isolation of high-quality RNA from about 5-fold more samples in the same time period. Notably, germinated seed was slightly easier to grind under Wang et al. [12] protocol due to the addition of sodium dodecyl sulfate (SDS) post grinding, which suppressed excessive foaming. The presence of denaturing β-mercaptoethanol in the buffer, used for tissue grinding without liquid N_2_ successfully inhibited RNase, as evidenced by RNA integrity (Figure 2). DNase I treatment removed the genomic DNA (gDNA) band (Figure 2). Also, no amplification in PCR with the gene specific primers confirmed DNase I treated RNA samples had no gDNA (Figure 3B).

We conducted quantitative real-time PCR (qRT-PCR) assays using cDNA synthesized from RNA extracted by the modified Li and Trick method, using several gene-specific primer sets relevant to barley malting quality characteristics. As expected, each primer pair amplified a single DNA fragment with the correct band size by reverse transcription PCR (RT-PCR) (Figure 3A), and produced a solitary peak in the melt curve of qRT-PCR (Figure 3B).

## 3. Conclusions

In conclusion, either the Li and Trick [11] or Wang et al. [12] methods, modified to extract RNA by direct grinding in buffer without liquid N_2_ are the simplest and fastest for a large number of germinated individual barley seed. These two protocols are nearly equivalent. Under either modified method, the resulting RNA samples are of sufficient quality for expression analysis of genes involved in germination and malting quality. This protocol was also effective for extracting RNA of germinated oat seed (data not shown) and will likely be effective for RNA extraction from germinated seed of other cereals that contain high amounts of polysaccharides.

## 4. Experimental Section

### 4.1. Preparation and Buffers

Prior to RNA extraction, all glassware, plastic pestles, and stainless-steel ware were rinsed with nuclease-free water and oven-dried. Other associated lab supplies, including microcentrifuge tubes, used in RNA extraction were purchased as nuclease-free.

### 4.2. Buffers (Previously Published in Li and Trick [11])

*Extraction buffer 1*: 100 mM Tris (pH 8.0), 150 mM LiCl, 50 mM EDTA (Ethylenediaminetetraacetic) acid, 1.5% sodium dodecyl sulfate (SDS), and 1.5% *β*-mercaptoethanol.

*Extraction buffer 2:* 70% guanidinium sulfate (*w*/*v*) (incorrect chemical reagent*), 750 mM sodium citrate (incorrect concentration*), 10% lauryl sarcosine (incorrect concentration*), and 2 M sodium acetate (incorrect concentration*). (*In our personal communication, the corresponding author (Harold Trick) of Li and Trick [11] indicated that they recognized the above errors after the paper was published and could not add an addendum).

*Correct extraction buffer 2: 4.2 M guanidine isothiocyanate, 25 mM sodium citrate, 0.5% lauryl sarcosine, and 1.0 M sodium acetate (Harold Trick, personal communication).

### 4.3. Modified from Li and Trick [11] Protocol*

(1) A single 3- to 4-day sprouted barley or oat seed was added to a 1.5 mL microcentrifuge tube with 400 μL of extraction buffer 1 and ground in solution with an RNase sterile micropestle.
(2) An amount of 250 μL of a phenol-chloroform mixture (1:1, pH 4.7) was added and centrifuged immediately at 21,000× *g* for 30 min at 4 °C.
(3) Approximately 250 μL of the top phase was transferred to a new tube.
(4) An equal volume of extraction buffer 2 was added, the tube was inverted to mix and incubated for 10 min at room temperature.
(5) An amount of 200 μL chloroform-isoamyl alcohol (24:1) was added and centrifuged as above.
(6) The supernatant was removed and added to 300 μL isopropanol and 250 μL 1.2 M sodium chloride in a new tube.
(7) The solution was mixed by inversion and placed on ice for 15 min, then centrifuged at 21,000× *g* for 30 min at 4 °C.
(8) The supernatant was decanted and the RNA pellet was carefully washed with 400 μL cold 70% ethanol.
(9) The RNA pellet was dried for 10–15 min in a laminar flow hood.
(10) The RNA was dissolved in 50 μL of nuclease-free water and used in downstream applications.

Refer to Wang et al. [12] for protocol. The same modification of grinding samples in solution with a RNase sterile micropestle was applied*.

### 4.4. RNA Analysis and Preparation

For spectrophotometric measurements, RNA concentration and purity were determined using a Take3 Micro-Volume PlateMicroplate with an Epoch UV-VIS spectrophotometer (BioTek Instruments, Inc., Winooski, VT, USA). For DNase digestion of RNA samples to remove contaminating gDNA and subsequent LiCl precipitation, the concentration was adjusted to 20–25 μg of total RNA in 50 μL nuclease-free water. Then, 6 μL 10× DNase I reaction buffer and 10 units total of DNase I (NEB Inc., Ipswich, MA, USA) were added. Samples were incubated for 60 min at room temperature. An amount of 60 μL of 5 M lithium chloride solution was added prior to incubation for 1 h to overnight at −20 °C. After centrifugation as above, samples were washed with 400 μL of cold 70% ethanol. The resulting DNA-free RNA preparations were air-dried as above, and resuspended in 40 μL of nuclease-free water. RNA was subjected to spectrophotometric measurements and agarose gel electrophoresis both before and after DNA-free treatments.

### 4.5. cDNA Synthesis and qRT-PCR

cDNA was synthesized, and RT-PCR and qRT-PCR were performed as described by Rashid and Deyholos [15]. The following gene-specific PCR primers were utilized to measure the corresponding genes (Fwd/Rev): *α-amyl1*, *alpha-amylase1* (M17126), (AACGAGAGCAAGCTGCAAAT/ATGAGGTTCCCCACATCGTA); *B-Hord*, *B-Hordein* (DQ267479), (AACCCCAAAAACAGTTGCAG/TGTGTGGTACCTGCTGTGGT); *Cellulose synthase A6*, *CesA6* (AY483155),(CAACAGCGGTTACCAATCCT/CCCTTGAGGAAGGGGTAGAG); *Cp3*, *Cysteine protease* (AB377533), (TGCTGCATCTACGAGTACGG/ATGAGGGCAGCAGCTGTAGT).

## Figures and Tables

**Table 1:** RNA quality and yield from the GeneJet, TRIzol, Li and Trick [11] and Wang et al. [12] methods. *Standard*: extraction after grinding in liquid N_2_; *modified*: extraction without liquid N_2_ by grinding in the extraction buffer; N/A; not applicable.

	Absorbance Ratios		
	A260/A280 *	A260/A230 *	
Extraction method	Before–After DNase I Treatment	Before–After DNase I Treatment	RNA per Seed μg/48 mg *
GeneJet Column	N/A	N/A	0.0
TRIzol method (standard)	1.85c–1.87b	0.57d–0.61c	1.1b
Li and Trick [11] (standard)	1.90bc–2.10a	0.80c–2.20a	12.9a
Li and Trick [11] (modified)	2.06a–2.05a	1.32a–2.12a	14.2a
Wang et al. [12] (modified)	1.99ab–2.08a	1.05b–1.86b	14.6a

* Bonferroni *t*-tests (Means with the same letter are not significantly different).
